# Validation of a Community-Based Approach Toward Personalized Dementia Risk Reduction: The Kimel Family Centre for Brain Health and Wellness

**DOI:** 10.14283/jpad.2024.98

**Published:** 2024-05-28

**Authors:** Nicole D. Anderson, D. D’Amico, S. Rotenberg, D. R. Addis, J. Gillen, D. Moore, J. A. Furlano, B. Tan, M. Binns, M. Santarossa, H. Chertkow, Canadian Consortium on Neurodegeneration in Aging (CCNA) CAN-THUMBS UP Study Group

**Affiliations:** 1 Rotman Research Institute, Baycrest Academy for Research and Education, 3560 Bathurst Street, Toronto, Canada M6A 2E1 Ontario; 2 Departments of Psychology and Psychiatry (Medicine), University of Toronto, Toronto, Canada; 3 Department of Occupational Therapy and Occupational Sciences, University of Toronto, Toronto, Canada; 4 Department of Psychology, University of Toronto, Toronto, Canada; 5 Department of Exercise Physiology, University of Toronto, Toronto, Canada; 6 Department of Family Medicine (Health Sciences), Queen’s University, Toronto, Canada; 7 Dalla Lana School of Public Health, University of Toronto, Toronto, Canada; 8 Department of Neurology (Medicine), University of Toronto, Toronto, Canada

**Keywords:** Dementia prevention, risk factors, lifestyle, multidomain intervention, aging

## Abstract

**Background/Objectives:**

The Kimel Family Centre for Brain Health and Wellness is a research-driven community centre testing the efficacy of personalized dementia risk reduction programming on dementia risk and cognition. The objective of this protocol is to validate this approach by following people for two years.

**Design/Setting:**

Participants will receive a comprehensive dementia risk assessment, including nonmodifiable and modifiable risk factors, from which they will receive a Personalized Dementia Risk Report and Program Strategy, indicating their health conditions increasing and their risk level in five modifiable risk domains: physical activity, brain-healthy eating, cognitive engagement, social connections, and mental wellbeing. Equipped with this information, participants will enroll in programs within the Centre to address their risk factors. Changes to their dementia risk, cognition, and Personalized Program Strategy will be communicated through re-assessments of risk factors every six months (risk and cognition) and every year (comprehensive assessment).

**Participants:**

Participants (n = 450) will be 50 years of age or older, without a diagnosis of dementia, and sufficiently fluent in English to complete the assessments and understand program instructors. One goal is that our participant sample will include people of low income (with fundraising providing free community centre membership), and from various ethnoracial backgrounds.

**Intervention:**

Participants will select programs to meet their Personalized Program Strategy. For physical activity, they will gradually work toward the Canadian Society for Exercise Physiology guidelines. For brain-healthy eating, they will learn about the Brain Health Food Guide and food label reading, and then take additional programs. For cognitive engagement and mental wellbeing, they will take at least one hour of relevant programming per week. Social connections will be reinforced throughout all programs. All participants will also have access to the Canadian Consortium on Neurodegeneration’s CAN-THUMBS Up online, educational program on modifiable dementia risk factors, called Brain Health PRO.

**Measurements:**

The comprehensive assessment includes numerous dementia risk factors, but the primary measures are risk in the five domains, health conditions proximal to those five risk domains, and cognition, and how these are affected by adherence and quality of goal-directed future simulation. We hypothesize a reduced risk in the five domains within six months, improvements in health biomarkers within a year, and maintenance of cognition within two years, with these benefits accruing with greater adherence, but only up to a point, at which benefits will plateau, and greater benefits among participants whose goal-directed simulations are more vivid, personally-relevant, achievable, and positive.

**Conclusions:**

This innovative approach overcomes a number of limitations present in prior multidomain dementia prevention trials. Adapting a preference clinical trial that is embedded in a community centre, where participants have autonomy to choose programs to address their modifiable dementia risk factors, has real-world applicability in the global effort to reduce dementia risk.

**Electronic Supplementary Material:**

Supplementary material is available in the online version of this article at 10.14283/jpad.2024.98.

## Introduction

**G**lobal dementia prevalence is expected to rise by 253% by 2050, relative to 2020, with a cost of 2.8 trillion USD (1). Brain pathology accumulates decades before dementia symptom onset (2), yet there are no medications to reverse the cognitive damage that dementia-causing diseases effect. At least 40% of global dementia cases can be attributed to twelve modifiable lifestyle factors (3). This percentage is likely higher, as that model did not include low socioeconomic status (4), low levels of cognitive engagement (5), high levels of stress (6), or unhealthy dietary patterns (7) (factors not included in the Lancet estimate), which are also linked to dementia risk. The need to address modifiable health and lifestyle factors to reduce dementia risk is urgent.

### Vision of the Kimel Family Centre for Brain Health and Wellness

We are taking an exceptionally innovative approach to dementia risk reduction in the Kimel Family Centre for Brain Health and Wellness (Kimel Family Centre), by running a research-driven community centre (https://kimelcentre.baycrest.org). Participants aged 50 years or older will receive a detailed dementia risk assessment and then will be given a data-driven personalized program strategy to address their risk in five domains: physical activity, brain-healthy eating, cognitive engagement, social connections, and mental wellbeing. With a few exceptions, participants will have autonomy in how they address a given domain. Moreover, participants can remain involved, with regular assessments, as long as they remain eligible. Validating our approach to multidomain lifestyle dementia risk reduction is a foundational step toward our long-term goal of significantly reducing dementia prevalence. In developing this approach we have been mindful of several limitations of existing dementia risk reduction clinical trials.

#### Offering personalized advice alone

Delivering personalized dementia risk-reduction goals with coaching resulted in greater cognitive improvement relative to a control in one study (8), but not another (9). Most adults know the importance of exercise, healthy diet, etc., but we need to go beyond advice (10) and address individuals’ capability, motivation, and opportunity to adopt healthy lifestyle behaviours (11).

#### Targeting a single risk factor

Many studies in this field focused on a single risk factor. Only some of these trials demonstrated benefits (10, 12, 13). For example, a recent intervention found null results on global cognition in those assigned to the MIND diet compared to a control diet (14). Other studies have found that addressing combinations of risk factors has greater cognitive benefits compared to addressing them in isolation, e.g. (15). Dementia risk factors need to be tackled in combination (10).

#### Randomizing intervention domains / Including individuals at low risk

Some studies have randomized participants to interventions. In the Synergic trial (15), individuals were randomized to various combinations of exercise, cognitive training, Vitamin D supplementation, and control conditions. However, there were no inclusion criteria for levels of physical and cognitive engagement. Gains identified in global cognition among those assigned to exercise, particularly when combined with cognitive training, may have been amplified had the study included only those not sufficiently engaged in physical and cognitive activities at study outset. Indeed, in the Multidomain Alzheimer Preventative Trial (MAPT (16)), greater improvements in global cognition in the multidomain intervention arm compared to the control arm became evident only in analyses restricted to those at high dementia risk due in part to lifestyle behaviours. Thus, interventions should be tailored to individuals’ specific constellation of risk factors.

#### Delivering interventions in a standardized way

Studies have also delivered their interventions in a standardized manner, instead of giving participants choice in how they address their risk factor(s). This approach neglects substantial individual differences in motivators and preference for different types of healthy lifestyle activities (17, 18). In our approach, participants will be given significant autonomy on how they address a given risk factor. We are adopting a pragmatic preference trial, as this approach enhances recruitment, compliance, retention, and real-world applicability (19).

#### Being time limited

Some multidomain interventions were arguably too short (10). For example, the MAX trial found no group differences in effects on global cognition, but trial participation was only 12 weeks (20). All of the studies described so far were discontinued at some point. Kimel Family Centre participants can continue to participate as long as they wish, as dementia risk reduction is presumably most effective with sustained healthy lifestyle behaviour engagement.

#### Ignoring individuals’ ability to imagine a healthier self

Dementia risk reduction trials have not considered the critical role that cognitive processes play in behaviour change. Relevant is goal-directed simulation, the intersection of intention (setting a goal), planning (organizing steps to reach the goal) and episodic simulation (imagining oneself in a future state) (21). Generation of vivid mental imagery about personal goals increases perceptions of likelihood of goal attainment (22), and if that imagery is positive, enhances motivation to achieve personal goals (23). Individuals whose goals were more attainable and personally-relevant, and who produced more vivid and positive goal-directed simulations not only had higher wellbeing and fewer depressive symptoms, but made more progress towards their goals at follow-up (21). In this study, we will investigate how goal-directed simulation predicts outcomes, preparing for future goal-directed simulation interventions to maximize behaviour change.

This study leverages the Canadian Consortium on Neurodegeneration in Aging’s (CCNA; https://ccna-ccnv.ca/) CAN-THUMBS UP (24); (https://www.canthumbsup.ca/) initiative in worldwide FINGERS multidomain dementia risk reduction collaboration (25). The funded Phase III of CCNA includes a six-month personalized online educational program about dementia risk reduction, Brain Health PRO (24), with 300 older adults. This will serve as a control for this study to address directly whether advice alone or implementation in a community-based setting is more effective at inducing healthy behaviour change.

### Validation Study Aims and Hypotheses

**Figure 1:**
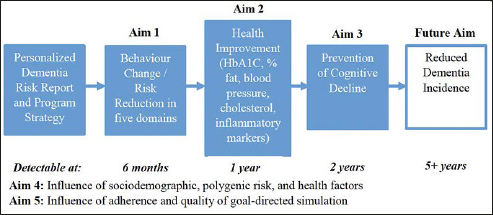
Validation study aims

We will follow 450 individuals (aged 50+) over two years, to identify the effects of an assessment-determined Personalized Dementia Risk Report and Program Strategy on dementia risk in our five domains, health biomarkers proximal to lifestyle behaviours, and cognition, as shown in Figure 1. Participants will sign up for programs to address their risk factors, with re-assessment and updated Personalized Dementia Risk Report and Program Strategy conveyed every six months. This pragmatic preference trial involves questionnaires pertaining to demographics, risk in the five domains, and health conditions. It is embedded within a larger, opportunistic study collecting data on a multitude of dementia risk factors (e.g., personality) intended for future research purposes.

#### Aim 1 Hypothesis

We expect our program to result in greater physical activity, brain-healthy eating, cognitive engagement, social connections, and mental wellbeing within the first six months compared to those who complete six months of Brain Health PRO alone (conducted by CCNA), and that this risk reduction will continue or be maintained over two years. In this study, we expect immediate uptake of behaviour change, because we are providing programs tailored to individuals’ capabilities and motivations, and are providing opportunity (the three factors of the COM-B model of behaviour change (11)) to engage in healthier behaviours. This hypothesis is supported by our finding a 21% increase in dietary adherence and large fitness improvements after a six-month diet and exercise intervention (26).

#### Aim 2 Hypothesis

Behavioural risk reduction is expected to result in improvement in health biomarkers proximal to behaviour change. We expect these changes to be detectable at the one-year mark, and then to have continued to improve at the two-year mark. This prediction is supported by similar results in the Finger trial (27) and by our trial which found substantial improvements in HbA1c (d = 1.02) after an exercise and diet intervention, compared to an active control (26).

#### Aim 3 Hypothesis

Behaviour change and health improvements are expected to result in maintenance of cognition, observable at the two-year mark, to the extent that participants adhere and have high quality goal-directed simulations (see Aim 5), which ultimately (but beyond the scope of this validation study) should result in lower dementia incidence.

#### Aim 4 Hypothesis

We will examine how sociodemographic factors and polygenic risk influence changes in behavioural risk reduction, health factors, and cognition, and how baseline health factors influence behavioural risk reduction and cognition. We do not expect these characteristics to affect outcomes, based on null results of sociodemographic factors in the FINGER trial (28) and comparable reductions in dementia risk associated with healthy lifestyle, irrespective of polygenic risk (29).

#### Aim 5 Hypothesis

We will examine how behavioural risk reduction, health factors, and cognition are influenced by participants’ adherence and quality of goal-directed simulations. We anticipate that benefits will accrue with greater adherence, but only up to a plateau, as found in reanalysis of MAPT results (30). We also expect greater benefits in people whose goal-directed simulations are more vivid, personally-relevant, achievable, and positive (21).

## Methods

### Ethics

This study is approved by the Baycrest Research Ethics Board (#23–26). All participants will provide written informed consent.

### Setting

The Kimel Family Centre is a 20,475 ft2 space of the Terraces, an assisted-living facility on Baycrest campus in Toronto. Like any larger community centre, it consists of a lobby with a reception desk, café-style and lounge-style seating; offices for full-time staff; two hoteling offices for volunteers and program instructors; a fully-equipped gym; a shallow, warm, salt-water pool with change rooms and showers; an activity room with soft flooring for floor exercises, but that can be repurposed as a classroom; another activity room for classes; a creative arts studio; an open-concept theatre; Research Hub A with two clinical exam rooms, a room with a pressure sensitive walkway, and a room with a treadmill and metabolic cart for fitness (VO2max) testing; and Research Hub B with a board room and five offices for researchers and trainees. The creative arts studio, theatre, and classrooms are equipped with a projector and screen, and all activity spaces have lockers for participants to store their belongings.

The Centre has eight staff members in addition to contracted program instructors. Trainees on student placements (e.g., kinesiology, nursing, occupational therapy) and a team of volunteers also provide support. The Kimel Family Centre is guided by two oversight committees comprised of Baycrest staff (e.g., Foundation and Finance staff) and older adult volunteers. A Business Advisory Committee oversees financial sustainability and provides budgetary guidance. A Visionary Advisory Committee oversees development and implementation of the model. The latter committee has sub-committees advising on participant, instructor, volunteer, and student experiences.

### Participants

Participants will be recruited by word of mouth, social media advertisements, community talks, and community ambassadors (described below). Recruitment conversations will begin with a study description and establishment of eligibility. Percentage of and reasons for non-recruitment (eligibility or barriers such as distance) will be recorded for each individual.

Participants will be eligible if they: (a) are 50 years of age or older, (b) are sufficiently fluent in written and spoken English to complete assessments and understand program instructors, (c) do not report a diagnosis of dementia, and (d) are willing to become members of the Kimel Family Centre at a fee of CDN $25 per month plus tax. Age and English fluency will be self-reported. If staff deem that a potential participant’s fluency is insufficient to meet eligibility criteria, they will provide information about other community centres in the area that provide programming in their primary language. There is a potential of undiagnosed dementia. Any participant who demonstrates signs of cognitive impairment (e.g., forgetting appointments, getting lost, confusion) and/or evidence of needing additional assistance (e.g., a care partner is needed for reasons other than mobility) will not be eligible, but will be given information about dementia-friendly community centres. The membership fee will subsidize costs of program staff, instructors, and supplies, but not research staff or expenses, which will be supported by donations and research grants. People with other conditions known to affect cognition (other than dementia), such as multiple sclerosis, traumatic brain injury, stroke, or mild cognitive impairment, will be eligible. Individuals who identify as Indigenous will be offered the opportunity to speak to Dr. Furlano for culturally appropriate assistance to interpret or support compliance with the study (31).

Our goal is for one third of our participants to be from low income households (with membership paid by our Links2Wellbing social prescribing grant [https://www.oacao.org/programs/links2wellbing/] and philanthropists), given the associated elevated dementia risk (4). The field is increasingly appreciating the need for an inclusive approach in research where diverse cultures are represented (32). Hence, another goal is that one third of our participants will come from various ethnic backgrounds. To achieve this, we are building relationships with community leaders to appoint community ambassadors, assessing the degree to which people from their culture would feel included, and advising on any changes or additions to our programming to implement. The remaining third of participants will be a sample of convenience. The first participant consented to participate in the study on March 25, 2024. We will recruit the first 450 participants by the end of the first year (10/wk), with data collection for this validation study taking three years.

### Assessments

At baseline and annually, participants will complete a comprehensive assessment of dementia risk and cognition, and annually, satisfaction with programming. Every six months between annual assessments, risk in the five domains, cognition, and satisfaction with programming will be assessed. The content of these assessments is described in Table 1.

**Table 1:** Schedule of Events

**Procedures**	**Baseline**	**6-Months**	**Annual**
BIOSAMPLE VISIT			
SALIVA [polygenic risk score (37)	X		
BLOODWORK [HbA1c, thyroid stimulating hormone, homocysteine, BDNF, triglycerides and cholesterols, c-reactive protein, interleukin-6]	X		X
CLINICAL VISIT			
HEIGHT/WEIGHT/BMI	X		X
SINGLE-TASK CONTROL FOR DUAL-TASK WALKING (53)	X		X
NASA/JSC PHYSICAL ACTIVITY SCALE [to estimate VO2max (PA-R; 54), along with sex, age, and percent body fat]	X		X
RESTING HEART RATE AND BLOOD PRESSURE [Welch Allyn Tycos]	X		X
BRIEF SMELL IDENTIFICATION TEST [Sensonics International]	X		X
MARS LETTER CONTRAST SENSITIVITY TEST [Mars Perceptrix]	X		X
MNREAD ACUITY CHARTS [Precision Vision]	X		X
GRIP STRENGTH [JAMAR Hydraulic Hand Dynanometer]	X		X
SHOEBOX AUDIOMETRY [Shoebox Limited; pure-tone hearing in each ear tested at 250, 500, 1000, 2000, 4000 Hz]	X		X
MONTREAL COGNITIVE ASSESSMENT (55)	X		X
ULTRASOUND [Phillips Affiniti 70; right quadriceps muscle]	X		X
COGNICITI BRAIN HEALTH ASSESSMENT (56)	X		X
SINGLE- AND DUAL-TASK GAIT (53)	X		X
DXA [General Electric Prodigy]	X		X
QUESTIONNAIRES VISIT			
SOCIODEMOGRAPHICS	X		x
SUBJECTIVE COGNITIVE DECLINE (51)	X		X
**COMMUNITY HEALTHY ACTIVITIES MODEL PROGRAM FOR SENIORS** (40) [CHAMPS; a measure of physical activity]	X	X	X
GET ACTIVE QUESTIONNAIRE (39) [to determine if medical clearance is needed for physical activity, and appropriate level of activity]	X	X	X
**FLORIDA COGNITIVE ACTIVITIES SCALE** (42) [plus added items asking about playing a musical instrument, volunteering, engagement in hobbies, and computer use]	X	X	X
**UCLA LONELINESS SCALE VERSION 3** (44)	X	X	X
**DEPRESSION, ANXIETY, AND STRESS SCALE** (45)	X	X	X
**PERCEIVED STRESS SCALE** (46)	X	X	X
**EATING PATTERN SELF-ASSESSMENT** (26) [measuring adherence to the Brain Health Food Guide, with the addition of caffeine intake (cups of coffee or tea a day)]	X	X	X
BIG FIVE INVENTORY-15 (57) [personality]	X		X
SENSE OF MEANING/PURPOSE IN LIFE (58)	X		X
CAREGIVER STATUS	X		X
FIRST DEGREE FAMILY HISTORY OF DEMENTIA	X		X
10-ITEM REVISED HEARING HANDICAP INVENTORY - SCREENING QUESTIONNAIRE (59) AND BRAIN-HEALTH PRO’S VISION AND HEARING RISK SCALE	X		X
HEAD INJURY HISTORY	X		X
ALCOHOL AND SMOKING	X		X
MEDICATIONS AND ADHERENCE	X		X
AMERICAN COLLEGE OF SPORTS MEDICINE CONDITIONS AND SYMPTOMS (60) [with added conditions queried]	X		X
MEDICAL CONDITIONS	X		X
MENTAL HEALTH HISTORY	X		X
SURGERIES IN LAST 10 YEARS	X		X
MENOPAUSE STATUS [females only]	X		X
FALLS WITHIN THE LAST YEAR	X		X
BRAIN HEALTH PRO’S SLEEP ASSESSMENT	X		X

Note: Bolded items are used to determine the Personalized Program Strategy. X = repeated in full; x = only items that can change will be repeated (specifically: gender, first three characters of postal code to extract satellite-based PM2.5 and NO2 from the Canadian Urban Environmental Health Research Consortium [https://canue.ca], current marital/partner status, live alone [binary], occupational status [working full time, working part time, looking for work, retired], annual household income [6 levels, plus I prefer not to answer]).

Raw data will be entered directly into REDCap, an electronic data capture platform (33, 34) hosted at the Rotman Research Institute. Other data types such as gait or DXA data will be stored in SPReD (35) comprehensive electronic databases. Automated data integration from these sources will leverage tools such as application programming interfaces and scripts written in R (36). De-identified databases will be backed up to an internal secured server daily, and a reproducible data pipeline written in R will be used to conduct data quality checks and calculate total (or component) scores for each measure, with code run when the full assessment is complete for a given participant.

#### Baseline Assessment

Participants will complete three baseline sessions in any order that suits their schedule.

#### Biosample Collection

Participants will visit Baycrest’s main campus to have 20 ml of random (non-fasting) blood drawn, spun, aliquoted, and frozen. Participants will also provide 1 ml of saliva. Blood and saliva will be shipped in batches of 94 to Sinai Health in Toronto to conduct blood analyses and DNA separation and genomic analysis using the Global Diversity Array chip. DNA single nucleotide polymorphism (SNP) results will be used to generate each participant’s polygenic risk score for Alzheimer’s disease (37). Because that approach is only validated for individuals of White European descent (37), DNA will also be stored in anticipation of more inclusive polygenic dementia risk scores.e.g. (38). Results will be received de-identified in csv format file for integration with the secure Kimel Family Centre database. Staff collecting these samples will be blinded to any aspect of participants’ dementia risk.

#### Clinical Assessment

Anthropometrics, sensorimotor health and functioning, body composition, and cognition will be assessed, as described in Table 1, in one session lasting approximately two hours. Although we are equipped for VO2max testing, this will not be included as a physician will not be on site. It is not feasible for the Senior Research Coordinator to remain blinded to participants’ dementia risk, as they will be conveying the risk report to participants.

#### Questionnaires

Participants will be escorted by a volunteer to an office in Research Hub B to complete questionnaires on their own on a tablet, in one session lasting approximately 60 minutes. The volunteer will be available should the participant have any technical difficulties. The questionnaires assess demographic information, subjective cognitive decline, lifestyle behaviours (detailed in Dementia Risk in Five Domains below), and a variety of conditions that are known dementia risk factors or that would inform what type and intensity of exercise participants can engage in safely. The Fitness Coordinator will be sent a list of these latter conditions, along with results of the Get Active Questionnaire (39). Importantly, we have mapped our risk assessment on to that of CCNA’s Brain Health PRO to allow a head-to-head comparison.

### Six-month Assessments

Every six months, the questionnaires informing the five dementia risk domains will be repeated, along with the Brain Health Assessment. The Get Active Questionnaire (39) will be repeated to keep participants’ fitness programs up to date. Participants will complete a questionnaire asking about satisfaction with different elements of participation, how much they feel they have learned about their dementia risk, and the importance of lifestyle for healthy brain aging. In the first completion of this survey, participants will be asked to reflect on their goals for becoming a member (method described in Goal-directed Simulation, below) and the degree to which they feel that their goals were met. Participants will be encouraged to describe what they like about the Kimel Family Centre and what could be improved.

Annual Assessments. Every year, the complete, three-session assessment and satisfaction survey will be repeated, except elements that do not change (e.g., genetics, date of birth).

### Personalized Dementia Risk Report and Program Strategy

Immediately after completion of the three baseline assessment sessions a Personalized Dementia Risk Report and Program Strategy will be generated electronically. This will contain the participant’s study identification number, but no identifying information, for privacy. The Senior Research Coordinator will meet with the participant to review this document.

#### Medical Conditions

The report will list any of the participant’s medical conditions that are known to associate with dementia risk. The Senior Research Coordinator will encourage the participant to follow up with their family physician (or walk-in clinic) on these conditions, to whom they will offer to write a letter. Resources will also be provided (e.g., flyers from the Canadian Heart & Stroke Foundation or Diabetes Canada).

#### Dementia Risk in Five Domains

**Figure 2:**
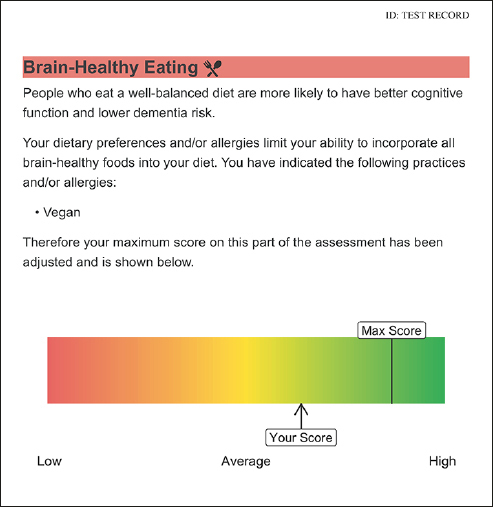
An example of how risk is conveyed in one of the domains For an example of a complete report, see Supplementary Material

The report will show the participant’s dementia risk level in five domains. For an example of risk in one of the domains, see Figure 2. Physical activity risk will be determined by the CHAMPS (40), which correlates with measures from activity monitors, whereas the PASE does not (41); brain-healthy eating risk by the Eating Pattern Self-Assessment, on which we demonstrated significant change after a diet intervention (26); cognitive engagement risk by the Florida Cognitive Activities Scale (42), which is negatively correlated with age and depressive symptoms, and positively correlated with education and cognition (43); social engagement risk by the UCLA Loneliness Scale Version 3 (44), and mental wellbeing by the subscales of the Depression, Anxiety, and Stress Scale (DASS-21 (45)) and by the 10-item Perceived Stress Scale (PSS-10 (46)). Loneliness was chosen, given that loneliness on the UCLA scale correlates with social network size and social support (44), and has a more consistent association with cognitive decline and dementia risk (47). The Stress subscale of the DASS-21 measures psychological distress (i.e., severe and/or prolonged stress), while the PSS-10 measures mild, moderate, and severe levels of perceived stress aligned with the Transactional Model of Stress and Coping (48). Including both the DASS-21 and the PSS-10 is important to capture a holistic view of stress as a risk factor for poor health outcomes, including dementia.

Risk in the physical activity domain will be defined as less than 150 min / wk of moderate-to-vigorous physical activity (i.e., not meeting CSEP guidelines). Risk in the brain-healthy eating domain will be less than a full score on the Eating Pattern Self-Assessment; for inclusivity, the maximum brain-healthy eating score will accommodate individuals’ dietary practices (e.g., vegetarian or vegan) or restrictions due to allergies. To determine risk in the mental wellbeing domain, we will apply DASS cut-offs (45) for mild depression (>9), anxiety (>7), and (dis) stress (>14), and the PSS-10 cut-off (46) for perceived stress (>13); scores exceeding one or more of these cut-offs will constitute risk in this domain. Normative cut-offs are not established for the Florida Cognitive Activities Scale or UCLA Loneliness Scale. Thus, we are conducting a normative study of people aged 50 years or older across Canada, with at least 100 participants per decade (50s, 60s, 70s, and 80s+), per sex, on these six questionnaires. Risk in the cognitive engagement and social connections domains will be defined as <91st percentile relative to these normative data. Based on preliminary data, we expect the following percentages of our sample to be at risk, per domain: physical activity: 42%, brain healthy eating: 100%, cognitive engagement: 90%, social connections: 90%, and mental wellbeing: 47%, with average number of domains at risk being ∼four.

#### Personalized Program Strategy

The report will provide participants’ Personalized Program Strategy. This will indicate which program domains participants should enrol in, and their strategic goal within that domain, as listed in Table 2. A specific strategy is not provided for social connections, as these will be promoted in all programs, as described in Kimel Family Centre Programs, below.

**Table 2:** Program Strategy per risk domain

**Domain**	**Program Strategy**
Physical Activity	Participants will meet with the Fitness Coordinator to develop a personalized plan to gradually get that participant exercising at least 150 minutes a week of moderate to vigorous activity a week, as well as strength training twice a week, and a focus on balance training for those over the age of 65, consistent with the Canadian Society for Exercise Physiology guidelines.
Brain-healthy Eating	Two required programs: one describing the Brain Health Food Guide, including how to adhere to it on a budget, and one on label reading. Take at least three hours of additional programming.
Cognitive Engagement	Take at least one hour a week of programming that is cognitively engaging (e.g., language learning, book clubs, creative arts).
Social Connections	No specific guidelines but social connections will be integrated into all other programs as well as special events.
Mental Wellbeing	Take at least one hour a week of stress reduction programming (e.g., yoga, meditation, spiritual classes).

### Goal-Directed Simulation

After participants review their baseline results with the Senior Research Coordinator, but before they select programs and take the SMART goal-setting workshop (see Kimel Family Centre Programs, below), participants will name goals they want to achieve to address each of their dementia risks (out of up to six: health conditions plus the five dementia risk domains) within six months. Participants will be instructed that goals should be personally relevant, achievable, and specific, with examples provided. Participants will select their most important goal per domain, and will rate each on dimensions such as perceived attainability, centrality to their identity, etc. Participants will then imagine and describe a specific future scene in their life related to each top goal, and will rate the vividness and emotional valence of their simulation (21).

### Bloodwork and Genetics Feedback

Results from blood work (annually) and genetics (baseline only) will be received a few months after collection. Participants will be able to opt out of learning their polygenic risk score. The Senior Research Coordinator will meet with participants to provide this information (per choice), emphasizing the beneficial effects of a healthy lifestyle on dementia risk, regardless of genetic risk (29).

### Updated Reports

Every six months, participants will receive an updated Personalized Dementia Risk Report and Program Strategy, and will be told if their risk in the five domains has changed. Our normative study is asking people to complete the six questionnaires a second time, seven to ten days later. From these normative data, we will calculate the minimal difference needed for a retest score to represent “real” change as an interval relative to a participant’s estimated true score T = μ + r(x − μ), where μ is the population mean and r is the reliability coefficient. The minimal difference will be determined as T ± 1.96 * SEP, where SEP is the standard error of prediction, defined as the SD of the first assessment * √1−r^2^ (49). A similar analysis is being conducted on existing data from the Brain Health Assessment. Any change greater than the minimal difference will be reported to participants as an improvement or decrement from the previous assessment.

### Kimel Family Centre Programs

Similar to a typical community centre, program offerings are organized by activity themes: arts and crafts; performing arts; lectures and continuing education; nutrition and cooking; mind, body, and soul; events, games, and social clubs; and fitness and aquatics. Some programs are offered virtually to maximize access. Staff and volunteers will be available to assist participants with program selection and participants will be encouraged to enroll in programs in domains in which they are not at risk (to remain at low or no risk).

All participants will complete a workshop series on SMART (Specific, Measurable, Achievable, personally Relevant, and Time-Specific) goal setting (50) at the start of their participation. The workshop will include one pre-recorded session about SMART goal setting and the rationale behind it. Additional bi-weekly group sessions, led by an occupational therapist, will be held to discuss and refine members’ goals relevant to their Personalized Program Strategy. If a participant has a dementia risk in the physical activity domain, the Fitness Coordinator will work with the participant to develop a plan that is appropriate for their fitness level/conditions and that matches their interests. There will not be any specific programming for those with social connections risk. Rather, all instructors will be trained to incorporate social connections within each of the programs (e.g., icebreakers, peer coaching and collaboration, and unstructured time for conversations in each session). Participants will also be paired with a Member Ambassador Volunteer – a buddy (who may or may not also be a participant) with whom they can discuss their experience and progress. In addition, special social events will be scheduled, such as a showcase event every term where participants are invited to share what they have created. In the brain-healthy eating domain, recipes that adhere to the Brain Health Food Guide but reflect the eating preferences of various cultures will be offered and co-developed in classes.

Mapping the five dementia risk domains onto each program offering was done by consensus by team members, based on scientific evidence. All programs are cross-listed in terms of the number risk domains they address (2–4 domains per program). The number of programs participants are asked to register for is therefore less than their total number of domains in which they are at risk. All participants will also be offered Brain Health PRO (24), tailored to their specific risk factors, to permit a head-to-head comparison of the effects of Brain Health PRO alone (CCNA) and Brain Health PRO plus hands-on programming on dementia risk reduction.

### Study Exit

Participants will remain in the study until they: a) voluntarily withdraw (with the reasons recorded), b) report a diagnosis of dementia, or c) have a decline of two standard deviations or more on the Brain Health Assessment and endorse needing support with activities of daily living (51), indicating dementia onset. Participants who develop dementia will be able to remain members (but not study participants), provided they are judged safe to do so with or without a care partner.

### Analysis Plan

We will conduct a series of hierarchical intent-to-treat linear mixed effect models to test our (i) whether behaviour (physical activity, brain-healthy eating, cognitive engagement, social connections, and mental wellbeing, with the latter being a composite of the DASS and PSS scales), health (HbA1C, % body fat, systolic blood pressure, total cholesterol, high density lipoprotein, BDNF, CRP, IL-6), and cognition (Brain Health Assessment) change during the observation period, then (ii) whether rate-of-change in the outcome measures associates with sociodemographic factors, polygenic risk, or clinical measures, and finally (iii) whether rate-of-change is modified by adherence or quality of goal-directed simulation. Specifically, the hierarchical model will include sequential blocks: (i) rate of change as a function of time with random intercept and slope (addressing Aims 1–3), (ii) sociodemographics (age at baseline, sex at birth, gender, years of education, living alone), polygenic risk score, and baseline health biomarkers (except for the biomarker outcome model) (addressing Aim 4), and (iii.a) linear and nonlinear effects of adherence (% attendance per domain) and interaction with time, or (iii.b) average rating of how vivid, personally-relevant, achievable, and positive the goal-directed simulations were and interaction with time (addressing Aim 5). The five behavioural/risk reduction models (Aim 1) will be rerun including CCNA participants who only completed the six-month Brain Health PRO study. This analysis will be restricted to baseline and six-month assessments. We will also explore relationships between adherence and goal-directed simulation quality, as individuals with a clearer, more positive image of their future selves may have greater adherence. Satisfaction data, changes in knowledge about dementia risk, and judgments pertaining to the importance of lifestyle for brain health will be analyzed in separate repeated measures ANOVA with four observations, both overall, as well as exploring the influence of sex, gender, age, and education. Goal attainment over the first six months will similarly be reported overall, and as a function of sex, gender, age, and education.

### Sample Size Calculations

We ran a simulation to estimate required sample size to test the full models described above. Based on statistics reported by Belleville et al. (30) we assume a normal distribution (M = 10, SD = 3) of sessions attended in a given domain and that baseline risk is associated with the number of sessions attended (r = 0.20). Trajectory of risk reduction was simulated from a limiting positive association with sessions (− k/sessions), a linear negative association with baseline risk, and random standard normal error. Additionally, we assumed a medium correlation with the number of sessions attended in an overlapping domain such as social engagement (r = 0.20). In order to have sufficient power to detect an adherence effect with the largest model, a linear regression model with 20 variables was fit to the trajectory data (baseline risk in the focal domain, focal domain adherence, reciprocal effect of focal domain adherence, adherence in the other four domains, five sociodemographic factors, polygenic risk, seven health factors). Accommodating potentially inflated error rate due to testing up to eight primary outcome measures (Aim 2), we reduced the alpha level for each of the models by a factor of eight (0.625%). We found 80% power to detect a medium-large dose effect (median f2 = 0.114, IQR = 0.085–0.147; comparable to our previous effect (26)) with 275 participants. Our first analysis will be restricted to the upper 85% of risk per domain, as those at the ceiling of behaviour are less likely to improve. This requires two-year data from 324 participants. Although we will follow an intent-to-treat approach, we anticipate a 15% attrition rate per year. We will thus need to recruit 448 participants, which we round to 450. The bottom 15% of risk per domain will be added back into the models to test whether even their behaviour can improve, resulting in health and cognitive benefits.

### Limitations

As innovative as the Kimel Family Centre is, there are limitations. This study only has a control group for the first six months, where changes in risk in the five domains will be compared to those in the CCNA-funded CAN-Thumbs UP study. For later time points, we are taking a dose-response approach, as a function of adherence and quality of goal-directed simulation. With the exception of retrospective self-reported questions, we are not capturing what people do outside of the centre in a comprehensive way. Grant applications are being prepared to add this. At present we can only give nutritional advice, or engage in minimal brain-healthy food preparation (e.g., with blenders or hot plates). There is space for a teaching kitchen, but additional fundraising is needed to renovate it. Requiring sufficient English fluency is necessary for valid data from the assessments and to maximize benefit from the programming; however, this does challenge our goal of cultural inclusivity.

### Implications

Dementia prevalence is going to rise exponentially in the coming years. In response, the Kimel Family Centre offers personalized multidomain programming that matches individuals’ improving capabilities, suits and strengthens their motivations, and provides opportunities to empower them to engage in healthy lifestyle behaviour needed to reduce dementia risk, improve health, and maintain cognition. Delaying dementia onset by just one year would result in half a million fewer cases in Canada by 2050 (52), which would have enormous financial and personal benefits. Acquired data will include all established or potential dementia risk factors. Other scientists and trainees will be able to leverage this richly phenotyped and growing (eventually −2000 participants) open database of longitudinal data. This trial has tremendous potential to generalize to other large community centres given their comparable facilities and program offerings. A community-based approach to dementia risk reduction is especially important to maximize reach and facilitate healthy behaviour change. Ultimately, this approach will help to cultivate healthy brain aging by reducing the incidence of dementia.

## Electronic supplementary material

Guide to your Dementia Risk Report and Personalized Program Strategy
