# Supplementary material for: Validation of a Community-Based Approach Toward Personalized Dementia Risk Reduction: The Kimel Family Centre for Brain Health and Wellness
Source: J Prev Alzheimers Dis. 2024 May 28;11(5):1455–66. doi: 10.14283/jpad.2024.98 (PMC11436454; doi:10.14283/jpad.2024.98)

Baycrest

KIMEL FAMILY CENTRE
for Brain Health and Wellness

# Guide to your Dementia Risk Report and Personalized Program Strategy

## How to read your report

This report summarizes the results of the data captured during your recent study visit. Included in this report is a list of addressable health conditions potentially contributing to dementia risk (**page 1**) and a Personalized Program Strategy that highlights your performance across 5 risk domains (**page 2**). These results are intended to help you better understand your own health and guide potential lifestyle modifications to optimize your well-being, with an emphasis on dementia prevention.

There are 5 risk domains we look at:

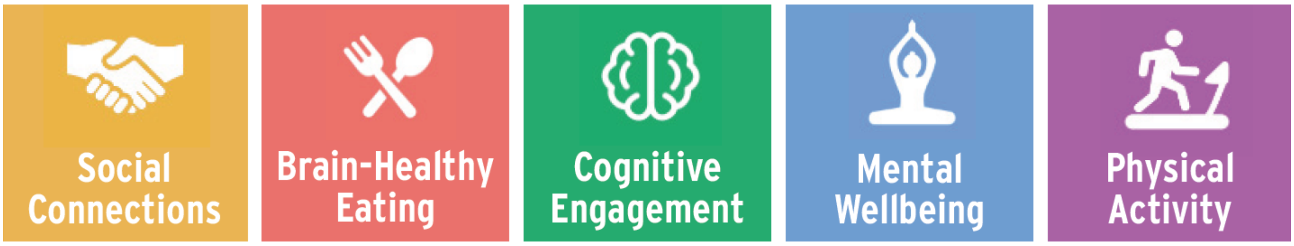


It is important to understand that the results presented in your report are modifiable and can change over time based on your lifestyle habits. For instance, if you find there are areas for improvement, consider how The Kimel Family Centre program guide can support your dementia risk reduction goals. If you are doing well in certain areas, reflect on how best to maintain your healthy behaviour and/or if there is room to progress.

## Your Scores

Your Personalized Program Strategy for each risk domain is derived from the questionnaires you completed when at the Kimel Family Centre. A breakdown of your scores can be found starting on **page 3**. The results have been translated to reflect where you stand based on pre-defined thresholds and relative to your demographics (age and sex). Please note that these scores are not diagnostic. Instead, use these scores to make informed decisions about your current lifestyle and as a baseline to track your progress when you receive your next report. Below is an example of what you can expect to see and how to interpret your results:

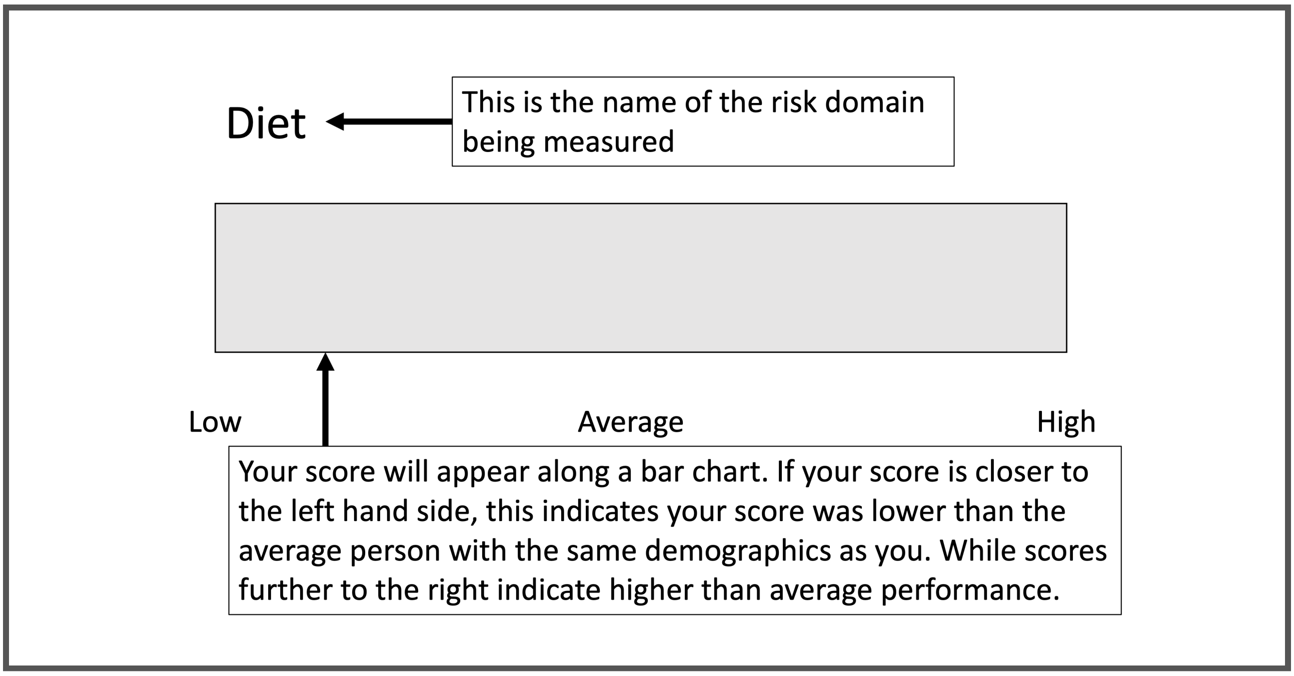


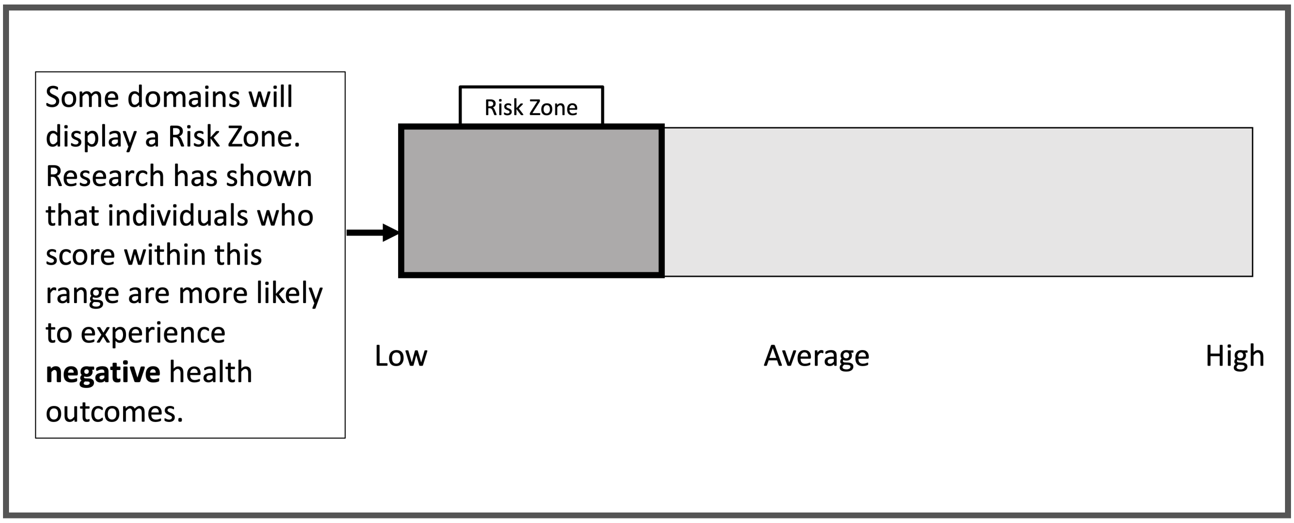

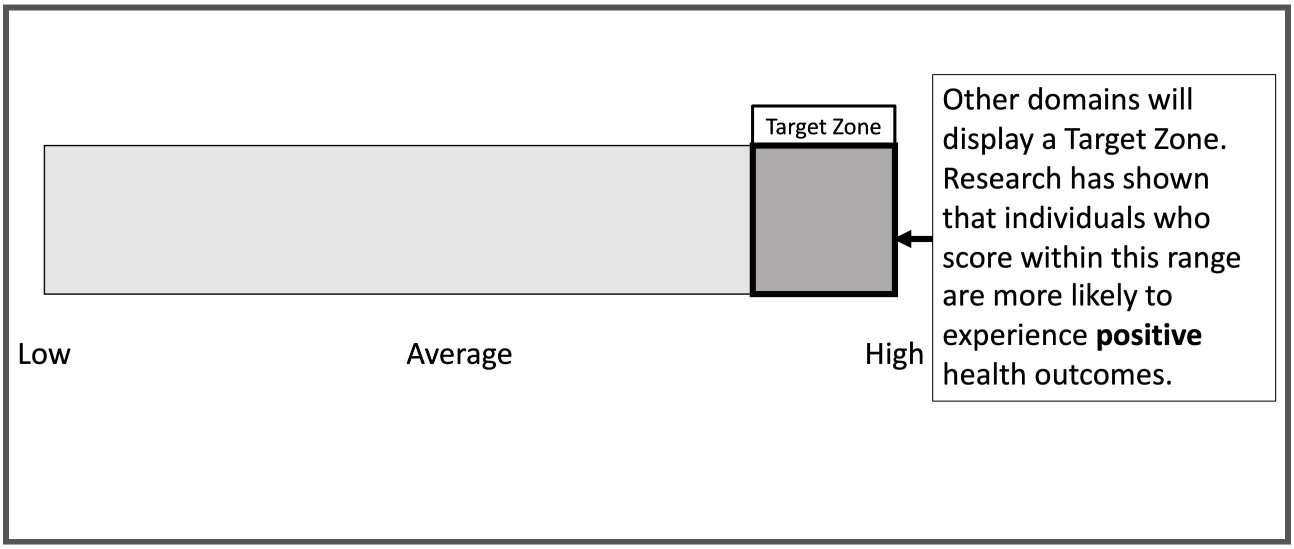


While we aim to provide you with information as accurate as possible, our measures may not have fully captured your unique lifestyle habits. What's important is that you take the results of this report and combine them with your own expectations to make informed decisions about how you manage your brain health.

## Next Steps

The research study does not enforce a strict regimen you must adhere to. While there are a few mandatory classes, how you manage your time and effort towards your dementia risk reduction on a day-to-day basis is up to you. Equipped with the information in this report, here are some general ways you can start making positive changes:

### Consult with the experts (including yourself)

- The Kimel Centre offers fitness consultations and general programming consultations with our staff to assist in meeting your goals. Please speak with the front desk for more information.
- Take some time to ask yourself if you are doing enough to address each domain. Where are you doing well? Is there room for improvement? Are there particular areas you want to focus on?
- Share your results with family, friends, and your health care professional. They may be able to support your efforts to reduce your dementia risk.

### Enroll in classes

- Programs and classes offered by the Kimel Centre are conveniently labeled with the same risk domain icons that appear in this report. Try your best to follow these recommendations and take programs related to your risk factors, but also choose programs that are of interest to you.

## Your Next Report

As per the study protocol, you will receive another report in approximately 6 months to track your progress.

KIMEL FAMILY CENTRE
for Brain Health and Wellness

# Your Dementia Risk Report and Personalized Program Strategy

Thank you for your participation in the Kimel Family Centre for Brain Health and Wellness study. This report contains results from your dementia risk assessment from **May 2024**. If you have any questions about your report, please reach out to one of our research coordinators or any member of our research team.

## Addressable Conditions and Other Factors Contributing to Dementia Risk

Some addressable conditions and/or other factors may be contributing to your risk for dementia. Please consider speaking to your healthcare professional on how to lifestyle modifications or medication may treat the following:

### Addressable Health Conditions

- Subjective Hearing Issues
- Subjective Vision Issues
- High Cholesterol

# Personalized Program Strategy

You are doing great in these areas, keep it up!

**Physical Activity**

**Cognitive Engagement**

**Mental Wellbeing**

Think about how you may wish to improve, if at all, in the following areas over the next six months to reduce your risk of dementia. Some areas may not need as much attention as others. Use the scores below to see how you're doing relative to other people your age.

**Social Connections**

All programs are designed to encourage social connections between participants and with instructors.

**Brain-Healthy Eating**

Take the two foundational courses, and then at least three additional one-session classes.

## Your Scores

### Cognitive Engagement

People who learn new information, practice skills and engage their mind are more likely to maintain their cognition with age. For example, engaging in arts and crafts, performing arts, and lecture series improves cognition.

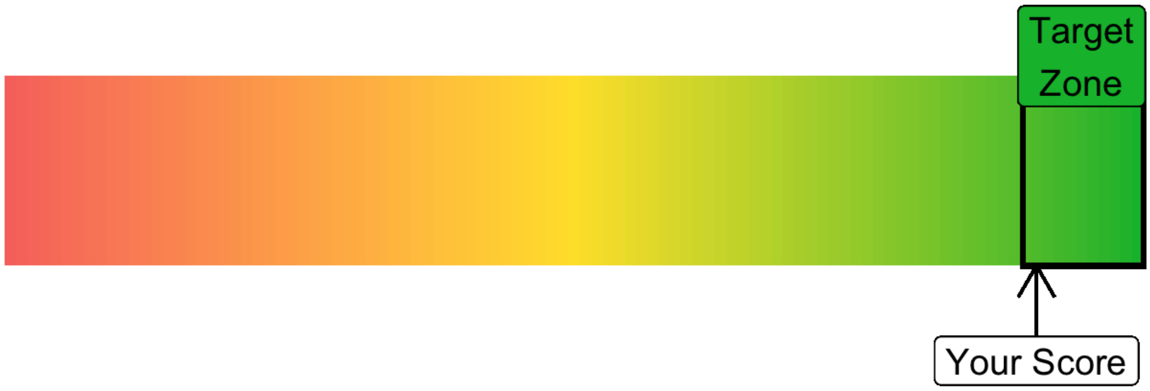


Low Average High

### Social Engagement

People who are socially connected are more likely to maintain their cognition with age.

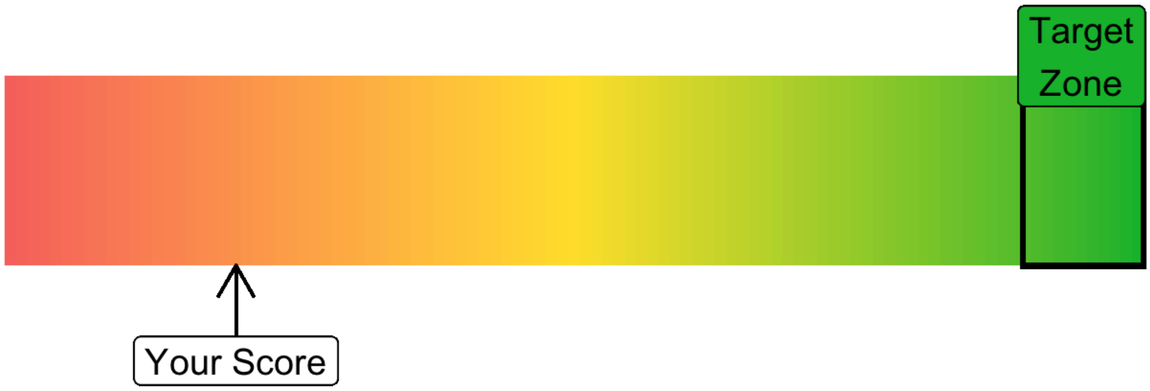


Low Average High

## Brain-Healthy Eating

People who eat a well-balanced diet are more likely to have better cognitive function and lower dementia risk.

Your dietary preferences and/or allergies limit your ability to incorporate all brain-healthy foods into your diet. You have indicated the following practices and/or allergies:

- Vegan

Therefore your maximum score on this part of the assessment has been adjusted and is shown below.

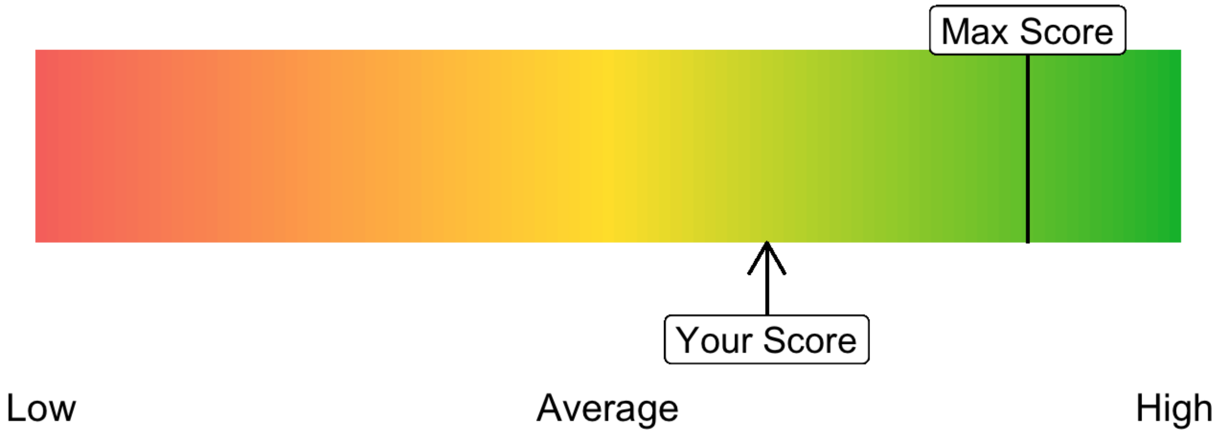

## Mental Wellbeing

Taking care of your mental health is beneficial to brain health. It is normal to experience some symptoms in the following areas some of the time.

## Symptoms of Depression

Symptoms of depression include hopelessness, low satisfaction with life, lack of interest or involvement, low energy, and difficulty feeling pleasure.

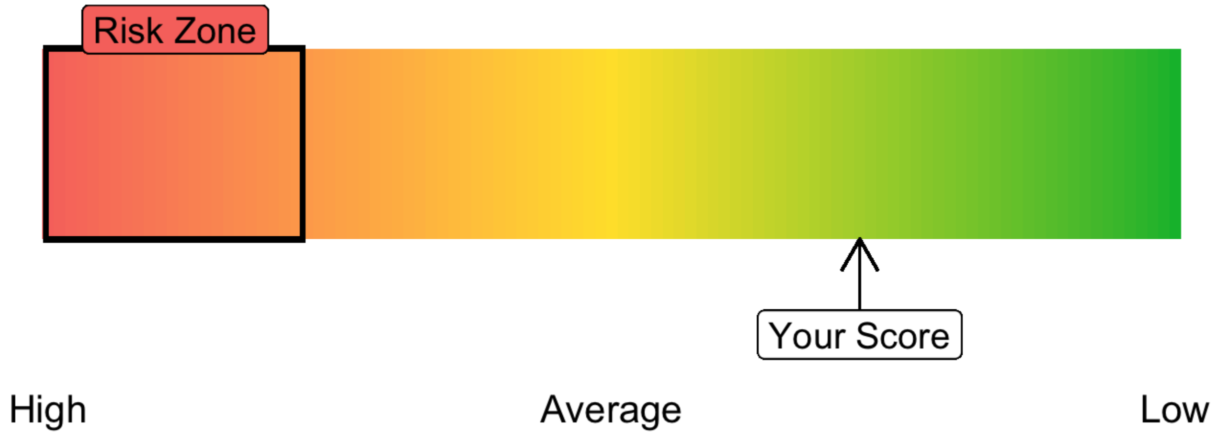


## Symptoms of Anxiety

Symptoms of anxiety include feelings of worry, panic, fear and/or feelings of nervous energy (such as trembling, increase heart rate).

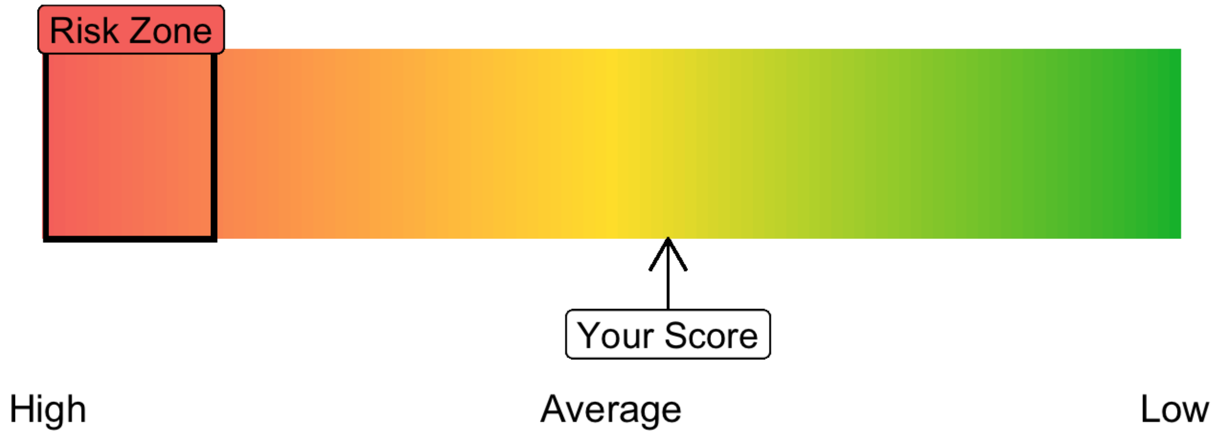

## Symptoms of Distress

Symptoms of distress include difficulty relaxing, being easily upset, agitated, irritable, overreactive, and impatient.

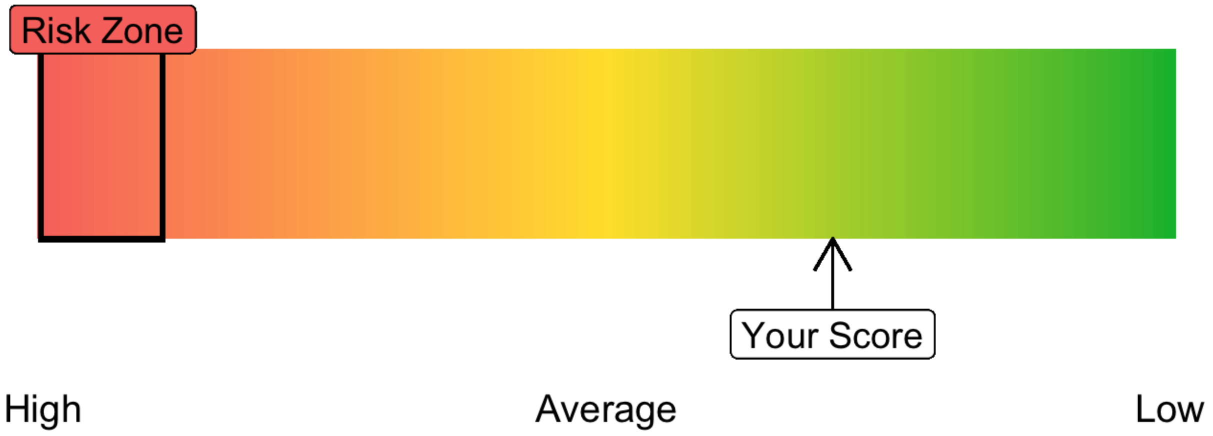


## Level of Perceived Stress

Level of perceived stress is based on your ability to cope with stresses within your life.

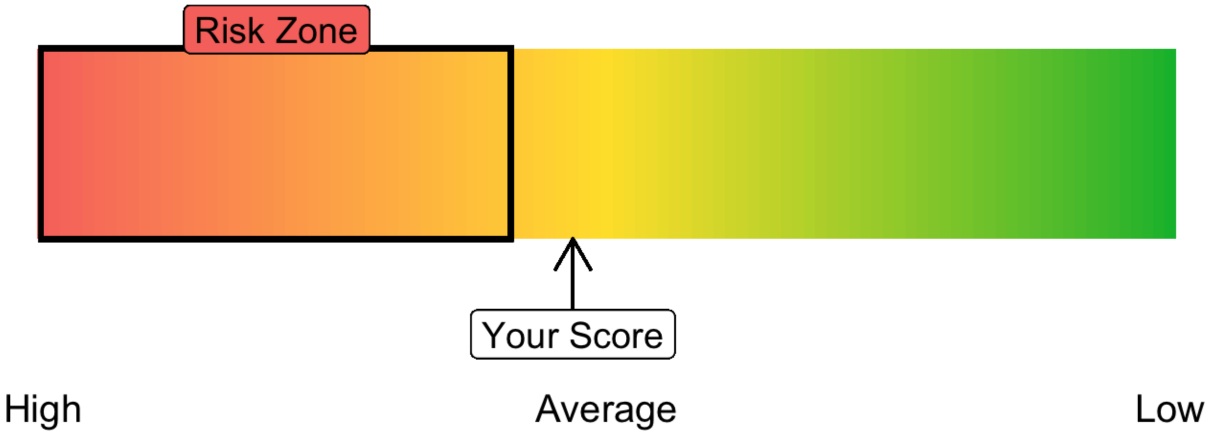

## Physical Activity

People who engage in physical activity are more likely to have improved cognition and mood, reducing their risk of dementia. It can even help to produce new brain connections from our learning and memory. The Canadian Society for Exercise Physiology recommends getting 150 minutes of moderate to vigorous activity a week.

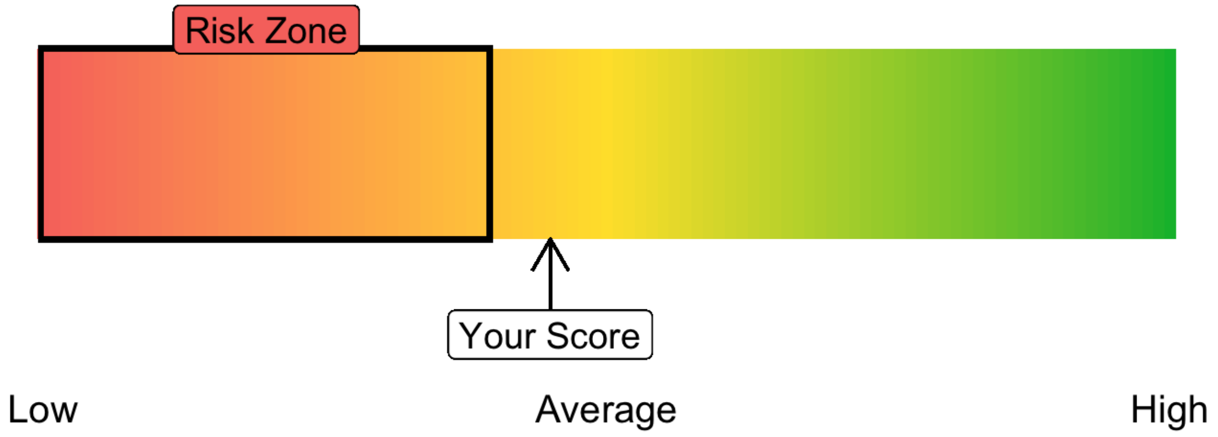

Supplement: Supplementary file 1 — Guide to your Dementia Risk Report and Personalized Program Strategy [file 42414_2024_98_MOESM1_ESM.pdf]
